# Early neonatal deaths with perinatal asphyxia of newborns ≥ 37-weeks in Brazil, 2000‒2020

**DOI:** 10.1016/j.clinsp.2025.100785

**Published:** 2025-09-23

**Authors:** Maria Fernanda B. de Almeida, Ana Sílvia S. Marinonio, Daniela T. Costa-Nobre, Mandira D. Kawakami, Adalberto O. Tardelli, Kelsy N. Areco, Paulo Bandiera-Paiva, Ruth Guinsburg

**Affiliations:** Escola Paulista de Medicina (EPM), Universidade Federal de São Paulo (Unifesp), São Paulo, SP, Brazil

**Keywords:** Infant, Newborn, Neonatal mortality, Developing countries, Epidemiological studies, Asphyxia neonatorum

## Abstract

**Objective:**

To evaluate temporal and geographical trends of early Neonatal Mortality Rate (NMR) associated with perinatal asphyxia in newborns ≥ 37-weeks’ gestation without congenital anomalies in Brazil, from 2000 to 2020.

**Method:**

The database was built based on live birth and death certificates, according to the municipality where birth occurred. Perinatal asphyxia was considered if ICD-10 codes were present in any line of the death certificate: intrauterine hypoxia (P20), birth asphyxia (P21) or neonatal aspiration of meconium (P24.0). Prais-Winsten method tested the temporal trend of early NMR associated with perinatal asphyxia per thousand live births. The geographical distribution was analyzed by municipality.

**Results:**

Over the 21 years, 55,204,633 live births were ≥ 37-weeks’ gestation without congenital anomalies, and 35,443 died with perinatal asphyxia up to day 7 of postnatal life. Early NMR associated with perinatal asphyxia decreased from 0.86 in 2000 to 0.45 in 2020 (annual change: -3.23%; 95% CI: -4.16% to -2.29%). This reduction was observed in all regions of the country, but North and Northeast showed, in 2020, rates above those found in 2000 in Southeast, South, and Midwest. The number of municipalities with early NMR associated with perinatal asphyxia >1.50 in Brazil decreased from 482 vs. 200 in 2000‒2005 vs. 2016‒2020.

**Conclusion:**

Since 2000, there has been a significant decline in early neonatal mortality rates associated with perinatal asphyxia among infants born at ≥ 37-weeks’ gestation without congenital anomalies in Brazil. However, in 2020, 0.45 per 1,000 of these live births still resulted in death, with marked regional disparities persisting.

## Highlights

• Most term infant deaths are preventable with improved perinatal care quality.
• Perinatal asphyxia is a key indicator of care quality for mothers and newborns.
• Early neonatal mortality associated with perinatal asphyxia has declined since 2000.
• Mortality is higher in the North/Northeast than in the South/Southeast of Brazil.
• Targeted interventions are urgently needed to reduce regional disparities.

## Introduction

According to the World Health Organization (WHO), approximately 6,300 newborns died daily in 2022, accounting for nearly 47% of all deaths among children under 5-years of age.^1^ The United Nations' Sustainable Development Goal 3.2 aims to end preventable deaths of newborns and children under 5, targeting a Neonatal Mortality Rate (NMR) below 12 per thousand live births in all countries by 2030.^2^ Brazil achieved this target in 2010, but regional disparities persist, with higher NMRs in the North and Northeast compared to the South, Southeast, and Midwest regions.^3^

The United Nations Inter-Agency Group for Child Mortality Estimation reported that Brazil's NMR declined from 18.91 per thousand live births in 2000 to 11.07 in 2010 and 8.84 in 2020, reflecting reductions of 41% between 2000‒2010 and 12% between 2010‒2020.^4^ However, the slower decline in recent years underscores the need for strategic planning to accelerate progress. Based on the five-phase neonatal mortality transition model, which classifies countries into stages ranging from NMR >45 (Phase I) to < 5 (Phase V), Brazil should aim to reduce its NMR to below 5 per thousand live births.^5^ Achieving this goal requires a thorough understanding of the causes of neonatal mortality.

Globally, the leading causes of neonatal mortality, prematurity, infections, and intrapartum-related events (perinatal asphyxia), are largely preventable, with congenital anomalies ranking fourth.^5^ In Brazil, population-based studies confirm that prematurity, congenital anomalies, perinatal asphyxia, and infections are the top causes of under-5 mortality, in descending order.^6^^,^^7^

Neonates born at 37‒41 weeks' gestation exhibit the lowest NMR in high-income countries. For example, in England and Wales in 2022, the overall NMR was 2.9 per thousand live births, dropping to 0.5 per thousand among term births.^8^ In Brazil, the corresponding rates in 2022 were 8.5 and 2.1 per thousand, with 70% of neonatal deaths occurring within the first week of life.^9^ Since most term neonatal deaths are preventable,^5^ and perinatal asphyxia is a key indicator of perinatal care quality, studying early neonatal deaths related to perinatal asphyxia can provide insights into barriers to improving care.

This study aims to evaluate temporal and geographical trends in early NMR associated with perinatal asphyxia in term newborns in Brazil from 2000 to 2020.

## Material and methods

This population-based study analyzed live births in Brazil from January 1, 2000, to December 31, 2020. It included neonates born at ≥ 37 weeks’ gestation without congenital anomalies who died with perinatal asphyxia up to day 7 of postnatal life.^10^ Brazil, classified as an upper-middle-income country, had a Gross Domestic Product per capita of US$10,043.60 and a Human Development Index of 0.760 in 2023.^11^^,^^12^ During the study period, Brazil's population grew from approximately 147 million in 2000 to 170 million in 2020. Regional population distribution remained consistent: North (8%), Northeast (28%), Southeast (42%), South (15%), and Midwest (7%).^13^ The number of births decreased from 3,206,761 in 2000 to 2,730,145 in 2020.^9^

The study used secondary data from the Brazilian Ministry of Health's Live Birth and Mortality Information Systems, which are publicly available in structured digital formats.^9^ Birth coverage increased from 93% in 2000 to 98% in 2020, and mortality coverage rose from 91% to 97% over the same period.^14^

Deaths were classified as associated with perinatal asphyxia if any of the following World Health Organization International Classification of Diseases, 10^th^ Edition (WHO-ICD-10) codes appeared in any line of the death certificate: P20.0 (intrauterine hypoxia first noted before labor), P20.1 (intrauterine hypoxia first noted during labor and delivery), P20.9 (unspecified intrauterine hypoxia), P21.0 (severe birth asphyxia), P21.1 (mild and moderate birth asphyxia), P21.9 (unspecified birth asphyxia), or P24.0 (neonatal aspiration of meconium).^15^ Deaths due to neonatal aspiration of non-meconium substances, neonatal cerebral depression, or fetal deaths were excluded. Congenital anomalies were identified using ICD-10 codes Q00–Q99 and excluded.^15^

The early NMR associated with perinatal asphyxia was calculated as follows: 1) Numerator: Number of neonates born at ≥ 37-weeks’ gestation without congenital anomalies who died with perinatal asphyxia up to day 7 of postnatal life; 2) Denominator: Number of live births at ≥ 37-weeks’ gestation without congenital anomalies. The ratio was multiplied by 1,000 to express the rate per thousand live births.^16^ Data were organized by the municipality of birth and study year.

Demographic variables extracted from death certificates included maternal age, maternal education, primiparity, delivery mode, infant’s sex, birth weight, place of death (hospital or other), and postnatal age at death. Analysis was performed for the following time periods: 2000‒2005, 2006‒2010, 2011‒2015, and 2016‒2020. The distribution of these variables across periods was assessed using the Chi-Square test for trends in SPSS (IBM SPSS Statistics for Windows, Version 21.0, IBM Corp., Armonk, NY).

Temporal trends in early NMR associated with perinatal asphyxia in Brazil and its five regions were analyzed using Prais-Winsten regression, which calculated the Annual Percentage Change (APC) and 95% Confidence Intervals (95% CI) with Stata 17® software (StataCorp LLC, Texas, USA).

The geographical distribution of early NMR associated with asphyxia by municipality was examined for the four time periods. Geographic files for Brazil and its municipalities were obtained from the Brazilian Institute of Geography and Statistics (IBGE).^13^ Municipalities were classified as those with ≤100 births during each period and those with 0‒< 0.5, 0.5‒< 1.0, 1.0‒< 1.5, or ≥ 1.5 early NMR associated with perinatal asphyxia per thousand live births. Data were imported into TerraView 4.2.2 (INPE, São José dos Campos) and visualized using thematic maps.

Ethical approval was waived by the Research Ethics Committee (CAAE 73319023.1.0000.5505) due to the use of secondary anonymized data from public sources, in accordance with Brazilian legal provisions regarding public domain works.

## Results

**Figure 1 Annual:**
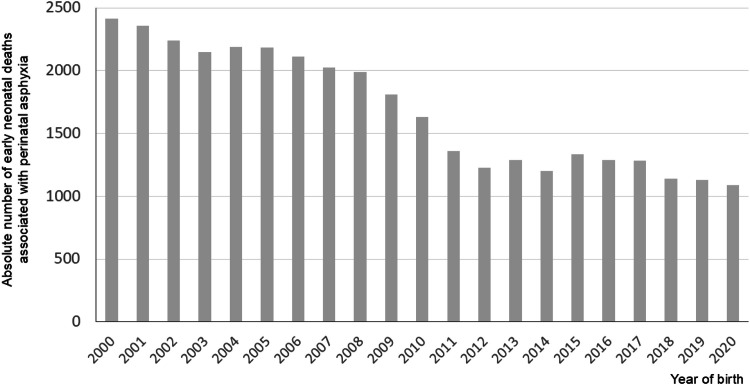
Number of early neonatal deaths of live births ≥ 37-weeks’ gestation without congenital anomalies associated with perinatal asphyxia, Brazil 2000‒2020.

Over the 21-year study period, 62,021,526 live births were recorded in Brazil. Of these, 55,204,633 were neonates born at ≥37-weeks’ gestation without congenital anomalies. Among this group, 35,443 deaths occurred up to day-7 of postnatal life with perinatal asphyxia (P20, P21, or P24.0) listed in any line of the death certificate. The annual number of early neonatal deaths associated with perinatal asphyxia is presented in Figure 1.

**Figure 2:**
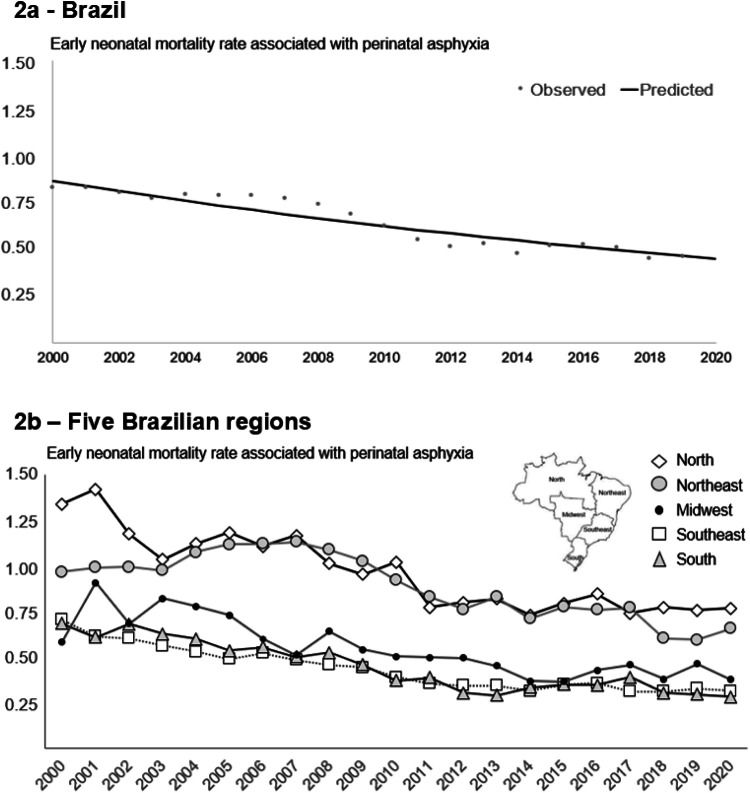
Annual early neonatal mortality rate with perinatal asphyxia per thousand live births ≥ 37-weeks’ gestation without congenital anomalies, 2000‒2020. (2a) Brazil (Prais-Winsten modelling). (2b) Five Brazilian regions.

The early NMR associated with perinatal asphyxia during the study period was 0.64 per thousand term live births. This rate declined from 0.86 in 2000 to 0.45 in 2020, with an APC of -3.23% (95% CI: -4.16% to -2.29%) (Fig. 2a). Prais-Winsten analysis revealed a decline in early NMR associated with perinatal asphyxia across all five Brazilian regions (Fig. 2b). The reductions were most pronounced in the South and Southeast regions, with the following APC values: North -3.04%; 95% CI: -3.83% to -2.25%; Northeast -2.43%; 95% CI: -3.84% to -1.00%; Southeast -3.94%; 95% CI: -4.89% to -2.97%; South -4.46%; 95% CI: -5.74% to -3.16%; and Midwest -3.74%; 95% CI: -4.79% to -2.29%.

Maternal and neonatal characteristics of the 35,443 deaths across the four study periods are shown in Table 1, revealing significant changes over time. Among infants born by Cesarean delivery, mortality declined from 0.93 per thousand live births in 2000 to 0.46 in 2020 (Prais-Winsten APC: -3.89%; 95% CI: -5.36% to -2.39%). For those born vaginally, the decline was from 0.74 to 0.48 per thousand live births (APC: -2.92%; 95% CI: -3.75% to -2.08%).

**Table 1:** Maternal and neonatal characteristics of infants ≥ 37-weeks’ gestation without congenital anomalies who died up to day-7 of postnatal life with perinatal asphyxia in four periods from 2000 to 2020 in Brazil.

	Number^a^	2000‒2005	2006‒2010	2011‒2015	2016‒2020	2000‒2020
**Age < 20 years**^a^	32,329	25.3%	24.1%	21.1%	18.0%	22.9%
**Schooling < 8-years**^a^	29,334	70.7%	58.7%	18.9%	10.6%	46.3%
**Primiparity**^a^	29,192	12.5%	21.0%	23.1%	22.6%	18.8%
**Cesarean delivery**^a^	35,063	44.1%	47.1%	53.0%	56.1%	48.6%
**Male**^a^	35,341	56.9%	55.7%	54.2%	53.1%	55.5%
**Birthweight ≥ 2500g**^b^	33,941	89.5%	89.1%	89.7%	91.1%	89.7%
**Hospital death**^c^	35,352	97.8%	96.9%	97.5%	97.3%	97.4%
**Death < 24h after birth**^a^	35,443	53.7%	40.3%	48.9%	45.8%	47.9%

Number of deaths with information available; Chi-Square for trend:

a p-value < 0.001;

b p-value = 0.002;

c p-value = 0.064.

**Figure 3:**
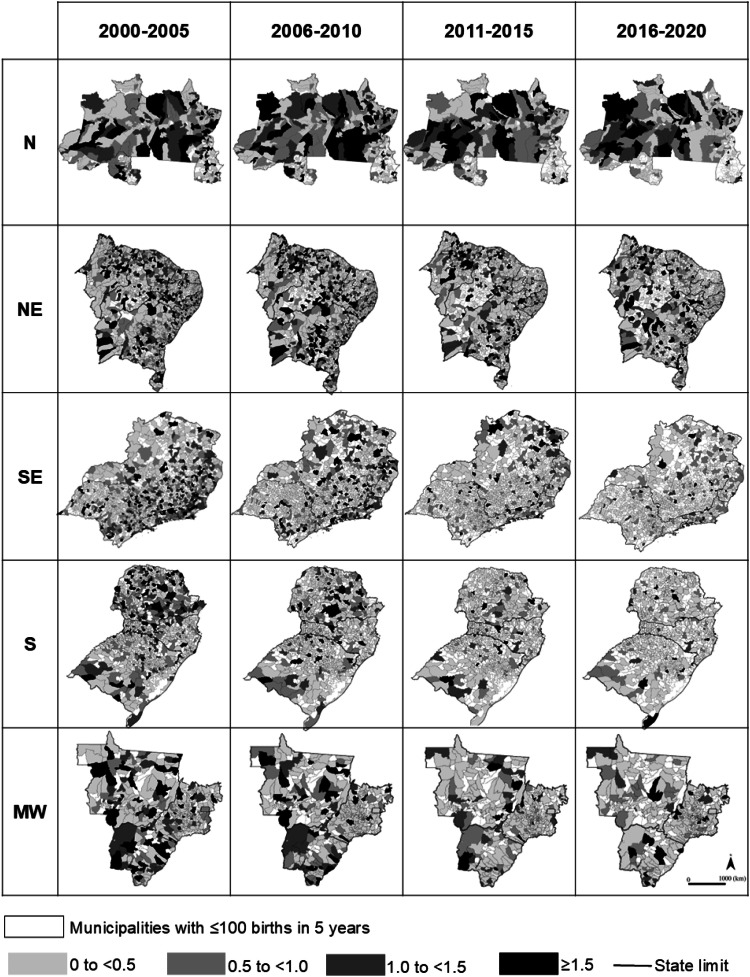
Geographical distribution of early neonatal mortality rate associated with perinatal asphyxia per thousand live births ≥ 37-weeks’ gestation without congenital anomalies per municipality in Brazilian regions (N, North; NE, Northeast; SE, Southeast; S, South; and MW, Midwest) in 2000‒2005, 2006‒2010, 2011‒2015 and 2016‒2020.

The geographical distribution of early NMR associated with perinatal asphyxia across Brazil's five regions during the four study periods is illustrated in Figure 3. Among Brazil's 5,570 municipalities, at least one death of an infant ≥37-weeks’ gestation occurred up to day-7 of postnatal life in 32%, 25%, 20%, and 18% of municipalities during 2000‒2005, 2006‒2010, 2011‒2015, and 2016‒2020, respectively.

Comparing 2016‒2020 to 2000‒2005, the reduction in the number of municipalities with at least one early neonatal death associated with perinatal asphyxia varied across regions: 21.4% in the North, 42.0% in the Northeast, 46.1% in the Southeast, 58.9% in the South, and 37.4% in the Midwest (Chi-Square for trend for each region, p < 0.001).

The number of municipalities with an early NMR associated with perinatal asphyxia ≥ 1.5 per thousand live births ≥ 37 weeks’ gestation decreased significantly, from 482 in 2000‒2005 to 200 in 2016‒2020 (Chi-Square for trend, p < 0.001).

## Discussion

Between 2000 and 2020, 35,443 neonates ≥ 37-weeks’ gestation without congenital anomalies died with perinatal asphyxia up to day-7 of postnatal life. The early NMR associated with perinatal asphyxia per thousand live births decreased from 0.86 in 2000 to 0.45 in 2020, reflecting an average annual reduction of 3.2%. Despite this decline across all regions, the North and Northeast exhibited higher rates in 2020 than the Southeast and South had recorded in 2000. It is worth noting that the decline in early NMR associated with perinatal asphyxia slowed in recent years, with rates stabilizing in 2018, 2019, and 2020, despite the emergence of the COVID-19 pandemic in Brazil in 2020.

The reduction observed in this study may be associated with improvements in population health. As highlighted by Massuda et al., between 2000 and 2014, total health expenditure in Brazil increased from 7.0% to 8.3% of the gross domestic product, while population coverage under the Family Health Strategy expanded from 7.6% to 58.2%.^17^ Additionally, federal and state governments implemented several national public policies focusing on maternal and neonatal care. These initiatives included the National Program for the Humanization of Pregnancy and Childbirth (2000),^18^ the Pact for the Reduction of Maternal and Newborn Mortality (2004),^18^ the creation of municipal and state committees to prevent infant mortality (2005),^19^ the Pact for Life (2006),^18^ the Stork Network (2011),^19^ and the Qualineo strategy (2017).^20^ These programs primarily sought to establish a network of health facilities to ensure that maternal care and childbirth occurred in settings with appropriate levels of complexity. They also emphasized improving healthcare providers’ competencies through training, mentorship, and continuous education initiatives.^21^^,^^22^ Although a causal relationship between these initiatives and the results obtained in the present study cannot be established, possible associations may explain the decrease in the early NMR associated with perinatal asphyxia.

There is evidence that socioeconomic factors significantly influence health outcomes.^23^ Individuals with lower socioeconomic status are at a higher risk of poor health, a pattern evident across Brazil's geographic regions. Brazil's gross domestic product increased from U$ 1,458 billion in 2000 to U$ 4,516 billion in 2010 and U$ 8,636 billion in 2020. The North and Northeast regions contributed 13%, 17%, and 18% to these totals, respectively.^24^ Despite economic growth in these regions during the study period, disparities remain. These disparities are evident in the available data on the number of obstetricians per 100,000 inhabitants and NICU beds per thousand live births across Brazil’s regions from 2008 to 2020. Although both indicators improved in all regions during this period, significant differences persisted. In 2020, the number of obstetricians per 100,000 inhabitants was: North 5.2; Northeast 7.3; Southeast 13.5; South 10.3; and Midwest 11.3. As for NICU beds per thousand live births in 2020, the figures were: North 1.8; Northeast 2.2; Southeast 4.6; South 3.6; and Midwest 3.3.^25^ Consequently, early NMR associated with perinatal asphyxia among infants born at ≥37 weeks’ gestation remains significantly higher in the North and Northeast regions (Fig. 2 and 3). Of the 200 municipalities with an early NMR ≥1.5 per thousand live births during 2016‒2020, 146 (73%) were located in these two regions. It should be noted that the cutoff of ≥1.5 per thousand live births was chosen because it exceeds the highest observed value of early NMR: 1.42 per thousand in the North region in 2001. Addressing regional and municipal-level inequalities is crucial to implementing targeted actions and programs that reduce socioeconomic disparities,^26^ improve health conditions, and prevent avoidable deaths.

The demographic characteristics of infants who died from perinatal asphyxia have evolved over the 21-year study period (Table 1). Specifically, the number of adolescent mothers decreased, and maternal education levels increased, reflecting improvements in the socioeconomic conditions of the Brazilian population. The Human Development Index of Brazil, for instance, rose from 0.679 in 2000 to 0.758 in 2020.^12^ Regarding delivery mode, while the Cesarean section rate is a key global indicator of access to obstetric services,^27^ the rates observed in this study likely reflect the high prevalence of this procedure in Brazil. The Cesarean section rate for infants born at ≥37-weeks’ gestation in Brazil was 39.7% in 2000‒2005, 48.0% in 2006‒2010, 56.2% in 2011‒2015, and 56.1% in 2016‒2020,^9^ which closely mirrors the rates reported in the present study. Also, these results showed that early NMR associated with perinatal asphyxia decreased for both delivery modes, with a more pronounced reduction among Cesarean section births. While confounding factors may exist, a comprehensive assessment of the impact of Brazil’s high Cesarean section rate on perinatal asphyxia mortality would require access to data on all live births, not just those resulting in death.

The observation that hospital deaths accounted for approximately 97% of the study population and remained unchanged over the years is not surprising. In Brazil, the proportion of hospital births for infants born at ≥ 37-weeks’ gestation was 96.9%, 98.2%, and 98.4% in 2000, 2010, and 2020, respectively.^9^ While deaths in the first days after birth comprised nearly half of early neonatal deaths associated with asphyxia in the studied infants, it is noteworthy that this proportion decreased over the study period. The reduction may be associated to the neonatal resuscitation training provided to Brazilian healthcare professionals, which may have contributed to fewer deaths in the delivery room. The Neonatal Resuscitation Program of the Brazilian Society of Pediatrics, launched in 1994, had trained 120,000 healthcare providers across all Brazilian federative units by the end of 2020. The rate of Brazilian-trained health professionals per thousand infants ≥ 37-weeks’ gestation increased from 17.9 in 2010 to 30.5 in 2020. In Brazilian regions, these rates were respectively in 2010 and 2020: North 9.5 and 29.2; Northeast 10.8 and 28.1; Southeast 27.5 and 39.8; South 13.5 and 17.8; and Midwest 13.5 and 17.8.^28^ The International Liaison Committee on Resuscitation strongly recommends accredited neonatal resuscitation courses for healthcare providers due to the consistent positive impact of this training, with the potential to save many lives.^29^ A meta-analysis by Patel et al., which included 20 trials involving 1,653,805 births in low- and middle-income countries, demonstrated that neonatal resuscitation training reduced the risk of 7-day neonatal mortality by 47% (RR = 0.53; 95% CI 0.38 to 0.73) compared to no training. Furthermore, the meta-analysis of pre- versus post-training mortality showed a decreased risk of 1-day neonatal mortality (RR = 0.58; 95% CI 0.42 to 0.82) and 7-day neonatal mortality (RR = 0.82; 95% CI 0.73 to 0.90) following training.^30^

The study has several limitations. The most significant is the use of secondary data, which may contain errors, underreporting, and a lack of contextual and clinical variables potentially associated with neonatal deaths, such as hospital complexity and staffing, maternal comorbidities, or access to neonatal resuscitation. The study utilized the Brazilian Live Birth Information System to calculate the number of newborns ≥37-weeks without congenital anomalies born each year in each municipality. However, the absence of a linked database of live births and neonatal deaths precluded conducting a multilevel regression analysis of variables associated with these deaths. It is important to note that the Mortality Information System in Brazil has undergone significant improvements in the vital statistics performance index through interventions aimed at increasing the completeness of death registration and training doctors in proper cause-of-death certification.^31^ Unfortunately, population-based studies like ours must rely on information systems that may oversimplify and misrepresent the true frequency of conditions such as perinatal asphyxia. As the only data source was death certificates in Brazil from 2000 to 2020, the authors adopted the best available approach: using the WHO classification for intrapartum-related neonatal deaths, which includes codes P20.0, P20.1, P20.9, P21.0, P21.1, and P21.9 for complications arising from intrapartum events. The authors also included code P24.0, for meconium aspiration syndrome, as a proxy for perinatal asphyxia, given its strong association with intrauterine hypoxia.^10^ Because codes P20.9 and P21.9 are non-specific regarding the timing and severity of the hypoxic/asphyxial insult, a sensitivity analysis was conducted, including only the 20,112 newborns whose death certificates listed, in any line, codes P20.0, P20.1, P21.0, P21.1, or P24.0. In this subgroup, early neonatal mortality associated with perinatal asphyxia declined from 0.43 per thousand live births in 2000 to 0.31 in 2020. Prais-Winsten analysis showed an APC of -2.92% (95% CI: -3.75% to -2.08%), consistent with the findings from the full cohort. Notably, the use of ICD-10 codes, which encompass intrauterine hypoxia, birth asphyxia, and neonatal aspiration of meconium, and considering these conditions beyond their classification as the basic or immediate causes of death, contributed to a better understanding of the role of perinatal asphyxia in early neonatal deaths in Brazil, particularly in infants who should not have died.

## Conclusion

Since 2000, there has been a significant decline in early NMR associated with perinatal asphyxia among infants born at ≥ 37-weeks’ gestation without congenital anomalies in Brazil. However, in 2020, 0.45 per thousand of these live births still resulted in death, with marked regional disparities persisting. To achieve a neonatal mortality rate of less than 5 per thousand live births, as outlined in the five-phase neonatal mortality transition model,^5^ priority must be given to reducing preventable deaths linked to intrapartum events in neonates ≥ 37-weeks’ gestation, particularly in the North and Northeast regions of the country.

## Ethical approval

The study was waived approval from the Research Ethics Committee (CAAE 73319023.1.0000.5505) due to the use of secondary anonymized data from a public source, in accordance with the provisions of the Brazilian legal system on public domain works.

## Funding

No funding was secured for this study.

## Consent statement

Patient consent was not required.

## Data availability statement

The database that originated the study is available with the corresponding author and may be freely retrieved from the following public source: Brasil. Ministério da Saúde. Datasus: Estatísticas vitais. Available at: https://datasus.saude.gov.br/informacoes-de-saude-tabnet/.

## Authors’ contributions

Maria Fernanda B de Almeida and Ruth Guinsburg participated in the concept and design; analysis and interpretation of data; drafting and revising the manuscript. Ana Silvia S Marinonio and Daniela T Costa-Nobre participated in the analysis and interpretation of data; drafting and revising the manuscript; Mandira Daripa Kawakami participated in the concept and interpretation of data; drafting and revising the manuscript; Adalberto O Tardelli, Kelsy VN Areco and Paulo Bandiera-Paiva participated in obtaining and consolidating the database and in revising the manuscript. All authors approved the final manuscript as submitted, and they agree to be accountable for all aspects of the work.

## Declaration of competing interest

The authors declare no conflicts of interest.
